# Direct comparison of the histidine-rich protein-2 enzyme-linked immunosorbent assay (HRP-2 ELISA) and malaria SYBR green I fluorescence (MSF) drug sensitivity tests in *Plasmodium falciparum* reference clones and fresh *ex vivo* field isolates from Cambodia

**DOI:** 10.1186/1475-2875-12-239

**Published:** 2013-07-12

**Authors:** Suwanna Chaorattanakawee, Stuart D Tyner, Chanthap Lon, Kritsanai Yingyuen, Wiriya Ruttvisutinunt, Siratchana Sundrakes, Piyaporn Sai-gnam, Jacob D Johnson, Douglas S Walsh, David L Saunders, Charlotte A Lanteri

**Affiliations:** 1 Department of Immunology and Medicine, Armed Forces Research Institute of Medical Sciences, Bangkok, Thailand; 2 Department of Emerging Infectious Diseases Program, US Army Medical Research Unit-Kenya, Kenya Medical Research Institute-Walter Reed Project, Kisumu, Kenya

**Keywords:** Immediate *ex vivo Plasmodium falciparum* drug susceptibility testing, HRP-2 ELISA, Malaria SYBR green fluorescence assay, Cambodia

## Abstract

**Background:**

Performance of the histidine-rich protein-2 enzyme-linked immunosorbent assay (HRP-2 ELISA) and malaria SYBR Green I fluorescence (MSF) drug sensitivity tests were directly compared using *Plasmodium falciparum* reference strains and fresh *ex vivo* isolates from Cambodia against a panel of standard anti-malarials. The objective was to determine which of these two common assays is more appropriate for studying drug susceptibility of “immediate *ex vivo*” (IEV) isolates, analysed without culture adaption, in a region of relatively low malaria transmission.

**Methods:**

Using the HRP-2 and MSF methods, the 50% inhibitory concentration (IC_50_) values against a panel of malaria drugs were determined for *P. falciparum* reference clones (W2, D6, 3D7 and K1) and 41 IEV clinical isolates from an area of multidrug resistance in Cambodia. Comparison of the IC_50_ values from the two methods was made using Wilcoxon matched pair tests and Pearson’s correlation. The lower limit of parasitaemia detection for both methods was determined for reference clones and IEV isolates. Since human white blood cell (WBC) DNA in clinical samples is known to reduce MSF assay sensitivity, SYBR Green I fluorescence linearity of *P. falciparum* samples spiked with WBCs was evaluated to assess the relative degree to which MSF sensitivity is reduced in clinical samples.

**Results:**

IC_50_ values correlated well between the HRP-2 and MSF methods when testing either *P*. *falciparum* reference clones or IEV isolates against 4-aminoquinolines (chloroquine, piperaquine and quinine) and the quinoline methanol mefloquine (Pearson r = 0.85-0.99 for reference clones and 0.56-0.84 for IEV isolates), whereas a weaker IC_50_ value correlation between methods was noted when testing artemisinins against reference clones and lack of correlation when testing IEV isolates. The HRP-2 ELISA produced a higher overall success rate (90% for producing IC_50_ best-fit sigmoidal curves), relative to only a 40% success rate for the MSF assay, when evaluating *ex vivo* Cambodian isolates. Reduced sensitivity of the MSF assay is likely due to an interference of WBCs in clinical samples.

**Conclusions:**

For clinical samples not depleted of WBCs, HRP-2 ELISA is superior to the MSF assay at evaluating fresh *P*. *falciparum* field isolates with low parasitaemia (<0.2%) generally observed in Southeast Asia.

## Background

*In vitro* testing of *Plasmodium falciparum* field isolates for susceptibility against currently applied anti-malarials provides an early warning of drug failure and possible clinical resistance. Several methods are commonly used to measure *P. falciparum in vitro* drug susceptibility, such as the schizont maturation test [1], [^3^H]-hypoxanthine incorporation [2], histidine-rich protein-2 enzyme linked immunosorbent assay (HRP-2 ELISA) [3,4], and most recently the malaria SYBR Green I fluorescence (MSF) assay [5,6]. The schizont maturation assay is based on microscopic examination for *P*. *falciparum* blood stage growth during drug exposure. Although relatively inexpensive, this assay is labour intensive and interpretation of results is subjective. The [^3^H]-hypoxanthine uptake assay involves measuring parasite growth by recording levels of hypoxanthine incorporated into parasite DNA [2]. While this method provides accurate and reliable results, the major drawback is the use of radioactivity, the safe disposal of which demands substantial resources. The HRP-2 method assesses parasite growth by using colorimetric ELISA to measure *P*. *falciparum* HRP-2 protein. This method offers a solution to examining drug susceptibilities of field isolates using a non-radioactive procedure that is also more cost effective compared to the [^3^H]-hypoxanthine method [3]. Recently, the MSF assay was developed based on measuring the incorporation of the fluorescent SYBR Green I dye into parasite DNA. This method relies on a single step of DNA staining, which is less labour intensive compared to an ELISA method, and is more amenable for high throughput screening of new drug candidates [7]. However, utility of the MSF assay in clinical isolates could be compromised in samples with a relatively low parasitaemia, because of non-specific fluorescence background attributed to SYBR Green I binding to human DNA of white blood cells (WBCs) that may reduce sensitivity of parasite detection [8].

Since 2004, Armed Forces Research Institute of Medical Sciences (AFRIMS) routinely applies the HRP-2 ELISA for “immediate *ex vivo”* (IEV) drug susceptibility testing of fresh *P*. *falciparum* field isolates without culture adaptation from multidrug resistant areas in Cambodia and Thailand [9-12]. This method generates IC_50_ results comparable to the World Health Organization (WHO) microplate schizont maturation test and the [^3^H]-hypoxanthine uptake assay, while offering the advantages of rapidity relative to the schizont maturation test and avoiding radioisotope use [4]. Moreover, measuring drug susceptibility of fresh parasite isolates without culture adaptation provides results that more accurately reflect the overall drug susceptibility phenotype of an *in vivo* infection by avoiding loss of drug-resistant parasite subpopulations during culture adaptation [13-15]. The HRP-2 ELISA performs robustly when analysing fresh isolates of relatively low parasitaemia. For example, in a previous related field investigation of malaria drug resistance, the HRP-2 assay had a success rate of 75% for IC_50_ value determination in samples collected during a survey in Cambodia and Thailand of 590 *P*. *falciparum* IEV isolates, of which nearly half had <0.2% parasite density [9].

Recently, several papers evaluated the utility of the MSF assay for measuring the drug susceptibility of *P*. *falciparum* reference and field isolates from Africa, where malaria transmission and thus patient parasitaemia are commonly higher than in Southeast Asia. The MSF assay produces comparable IC_50_ results to those generated by traditional [^3^H]-hypoxanthine incorporation and HRP-2 ELISA methods [5,6,16,17], but presumably only when parasitaemia is sufficiently high to produce reliable results. For example, a study conducted in Madagascar reports that the MSF method works well, with an overall 80% success rate of generating IC_50_ results when testing fresh *P*. *falciparum* samples of least 0.1% parasitaemia and washed three times prior to plating [17]. Another report [16] using fresh isolates from non-immune travellers who acquired malaria in West Africa indicates that the MSF assay yields the same success rate as the HRP-2 ELISA method when analysing samples with a mean parasitaemia of 0.75%, also washed three times prior to plating. In a recent investigation [6] of IEV isolates collected from western Kenya, the MSF assay produced a success rate of 78% with a parasitaemia >0.5%, whereas the success rate decreased to only 61% in samples with ≤0.5%.

However, the MSF assay is not expected to perform well to evaluate field isolates in whole blood samples collected from patients with lower parasitaemia. For example, a comparison of the MSF and HRP-2 ELISA methods for measuring *in vitro* susceptibilities of *P*. *falciparum* laboratory reference strains showed that the HRP-2 assay provides a similar limit of detection in either whole blood-media mixtures or WBC-free samples, whereas sensitivity of the MSF assay diminishes greatly in samples spiked with WBCs as a result of confounding fluorescence background signal [8]. This confounding effect, likely a result of SYBR Green I binding to WBC DNA, is further problematic in *P*. *falciparum* reference samples of lower parasitaemia <1.0% [8], as is typically encountered in regions of low malaria transmission. To date, a direct comparison of MSF *versus* HRP-2 methods has not been conducted using fresh clinical isolates of *P*. *falciparum* with such low parasitaemia.

The study described herein was conducted to evaluate utility of the HRP-2 ELISA *versus* MSF methods for determining *ex vivo* drug susceptibility of fresh *P*. *falciparum* isolates from Cambodia, representing an area of relatively low parasite transmission and thus low parasite density in patients. The hypothesis was that the HRP-2 ELISA would be more sensitive than the MSF assay when testing fresh clinical isolates of low parasitaemia (<0.2%).

## Methods

### *Plasmodium falciparum* culture and synchronization

*Plasmodium falciparum* reference strains W2, D6, 3D7, and K1 were maintained as described previously [18]. Briefly, cryopreserved vials of reference clones were recovered using 3.5% sodium chloride [19] and maintained in RPMI-1640 medium containing 25 mM HEPES, 25 mM sodium bicarbonate, 5% human O^+^ erythrocytes, 10% pooled AB^+^ serum and 0.1 mg/mL gentamycin at 37°C with 5% CO_2_, 5% O2, and 90% N_2_. To obtain a predominance (≥90%) of ring stage parasites for drug susceptibility testing, 5% D-sorbitol synchronization was performed as previously described [20,21]. After synchronization, parasites were maintained for 48 hours prior to conducting the drug susceptibility assay.

### *Plasmodium falciparum* isolate collection and sample processing

Clinical *P. falciparum* isolates were obtained from patients with uncomplicated falciparum malaria enrolled in an anti-malarial drug resistance surveillance study in Cambodia. The study was approved by the Cambodian National Ethics Committee for Health Research (NECHR), and the Walter Reed Army Institute of Research (WRAIR) Institutional Review Board (protocol number: WRAIR 1576). After signing informed consent, malaria patients ≥ 13 years old without a history of anti-malarial drug use within the previous seven days were enrolled into the study. Diagnosis of malaria was conducted using Giemsa-stained peripheral blood smears, with microscopy species determination verified using real-time PCR targeting the 18 s rRNA as reported elsewhere [9]. A total of 41 isolates, collected from volunteers with mono *P. falciparum* infection living in northern (Preah vihear province) and southern Cambodia (Kampong Spou, Kampot, and Preah Sihanouk provinces) during May -October 2011, were evaluated in the HRP-2 and MSF assays. At the time of diagnosis (before treatment), patient blood samples (4 ml of venous blood in sodium heparin tubes) were tested for IEV drug susceptibility, without a leukocyte depletion step or culture adaptation, as previously described [9]. Samples were applied to dried drug plates for HRP-2 and MSF assay analysis within six hours after phlebotomy.

### Preparation of dried drug plates

Dried drug plates, for use in the HRP-2 and MSF assays, were prepared using published methods [9]. Briefly, six drugs, dihydroartemisinin (DHA), artesunate (AS), mefloquine hydrochloride (MQ), quinine sulphate hydrate (QN), chloroquine diphosphate (CQ), and anhydrous piperaquine phosphate (PPQ), were coated onto 96-well plates in duplicate. Molecular weights of DHA, AS, MQ, QN, CQ, and PPQ used were 284.35, 384.42, 414.78, 782.97, 515.92, and 927.57 g/mol, respectively. All test drugs were provided by the WRAIR (Silver Spring, MD, USA). Drugs were dissolved in 70% ethanol to make 1 mg/ml stock solutions, with the exception of using 0.5% lactic acid in distilled water as a solvent for PPQ. The drug stock solutions were then diluted to appropriate concentrations in sterile distilled water. Three-fold serial drug dilutions were performed on plates to reach final concentrations (after 200 μL of sample added) ranging from: 0.027 to 20 ng/ml for DHA and AS, 0.274 to 200 ng/ml for MQ, 1.71 to 1,250 ng/mL for QN, 2.74 to 2,000 ng/ml for CQ, and 0.86 to 625 ng/mL for PPQ. The top row of each plate served as a drug-free control for parasite growth. Drug plates were dried overnight in a running biosafety cabinet and stored at 4°C up to eight weeks prior to use. As a quality control for dried drug plate integrity during transport to and from field sites, a subset of plates not used in the assays were tested to ensure an acceptable range of IC_50_ values was attained against the *P*. *falciparum* W2 reference clone, as described previously [21].

### HRP-2 ELISA and MSF *Plasmodium falciparum* susceptibility testing

Performance of HRP-2 ELISA and MSF methods was evaluated side-by-side in *P*. *falciparum* reference lines and in Cambodian IEV isolates. Each reference clone or fresh isolate sample was added to the same drug-coated plates, which were later processed for each assay as described below. Synchronized cultures of *P*. *falciparum* reference strains with ≥90% ring forms were diluted to 0.5% parasitaemia with 1.5% haematocrit in 0.5% Albumax RPMI 1640, and transferred to dried drug-coated plates. Plates were incubated at 37°C with 5% CO_2_, 5% O_2_, and 90% N_2_ for 72 hours. For IEV drug susceptibility testing, blood samples with ≤0.5% parasitaemia were transferred to dried drug-coated plates without sample dilution and those with >0.5% parasitaemia were diluted to 0.2-0.5% prior to plating. Plates containing IEV isolates were incubated at 37°C using a candle jar, instead of mixed gas.

After 72 hours of incubation, plates were frozen and thawed and then analysed in parallel for parasite growth inhibition by HRP-2 and MSF assay. For HRP-2 assay analysis, culture samples were diluted before performing ELISA as described in Rutvisuttinunt *et al.*[21] and Tyner *et al.*[9]. The MSF assay was performed according to Johnson *et al.*[5]. Briefly, for the MSF assay 100 μL of samples per well were transferred to Optiplate-96 F black plates (Perkin Elmer, Salem, MA, USA) prior to adding 100 μL lysis buffer [20 mM Tris (pH 7.5), 5 mM EDTA, 0.008% (wt/vol) saponin, and 0.08% (vol/vol) Triton X-100] containing SYBR Green I (1X final concentration). Plates were incubated for one hour in the dark at room temperature and then relative fluorescence unit (RFU) measurements in each well were made using a Victor plate reader (Perkin Elmer, Salem, MA, USA) at an excitation/emission wavelength of 485/535 nm. HRP-2 optical densities (OD) and SYBR Green RFU of duplicate wells were averaged and plotted against drug concentrations. IC_50_ values were estimated by nonlinear regression analysis using the ICEstimator program [22].

### Assessing SYBR Green fluorescence linearity and detection limit for parasitaemia

SYBR Green fluorescence linearity of parasitaemia between 0-10% was examined by serially diluting synchronized *P. falciparum* ring forms of strains 3D7 and K1 with non-infected erythrocytes as described previously [5]. Briefly, two-fold serial dilutions of parasite cultures were performed using non-infected erythrocytes with 1.5% haematocrit in culture medium. Then 100 μL cell media mixture with varying levels of parasitaemia and non-infected erythrocyte samples were added in 12 replicate wells in each row of the 96-well plates. Next, 100 μL lysis buffer containing SYBR Green I dye (1X final concentration) was added before incubating plates for one hour in the dark at room temperature, followed by taking RFU measurements as previously described. To assess fluorescence linearity in *P. falciparum* samples spiked with WBCs, similar procedures were done, but using only 50 μL of samples with varying parasite densities and non-infected erythrocyte samples in 12 replicate wells of each row. Next 50 μL of 1.5% haematocrit blood containing 17,000 WBC/μL was added to yield samples with 0-5% parasitaemia containing 8,500 WBC/μL, representing maximum WBC counts observed in malaria patients in Cambodia [10]. After subtraction of non-infected erythrocyte background, average RFUs of 12 replicate wells were plotted on the Y axis against % parasitaemia on the X axis. Linear regression line, r^2^, limit of detection (LOD), and limit of quantitation (LOQ) were determined using MS Excel. Up to three experiments were conducted to yield the LOD and LOQ range, calculated as per International Conference on Harmonization (ICH) guidelines using the equations below.

$$\begin{array}{ll} \mathrm{LOD}= & 3*\mathrm{the}\;\mathrm{residual}\;\mathrm{standard}\;\mathrm{deviation}\\ & /\mathrm{slope}\;\mathrm{of}\;a\;\mathrm{regression}\;\mathrm{line}\\ \mathrm{LOQ}= & 10*\mathrm{LOD}/3 \end{array}$$

### Reliability of HRP-2 and MSF drug susceptibility assays in samples with low parasitaemia and effect of % parasitaemia on IC_50_ values

To examine performance of HRP-2 ELISA and MSF drug susceptibility methods in samples with low parasitaemia, *P. falciparum* W2 and D6 parasites were applied to dried drug plates at varying parasitaemia of 0.005, 0.01, 0.05, 0.2, and 0.5% and the assays were conducted as described above. Two experiments were performed for each reference strain. The lowest tested parasitaemia that produced best-fit sigmoidal concentration-response curves resulting in IC_50_ values was determined for each method. In addition, IC_50_ values obtained from varying parasitaemia were compared to investigate the effect of baseline parasitaemia (inoculum effect) on IC_50_ values.

For IEV drug susceptibility testing, reliability of the HRP-2 and MSF methods in samples with low parasitaemia was evaluated as the assay success rate when testing DHA, AS, MQ, QN, CQ, and PPQ activities. A “successful” IC_50_ assay result for each *P. falciparum* clinical isolate was defined as achieving a sigmoidal concentration-response with an IC_50_ confidence interval ratio ≤ 5 when testing six serial drug dilutions for at least one of the tested drugs.

### Statistical analysis

IC_50_ values obtained from HRP-2 ELISA and MSF methods were compared using Wilcoxon matched pair tests. Log transformed IC_50_ values were utilized to analyse the correlation and agreement between the two methods using Pearson’s correlation and Bland-Altman plots, respectively [4,23]. The Kruskal-Wallis test was performed to examine the effect of baseline parasitaemia on HRP-2 ELISA IC_50_ values. Statistical analysis was performed using GraphPad Prism (GraphPad Software, Inc, San Diego, CA, USA).

## Results

### SYBR green fluorescence linearity

**Figure 1:**
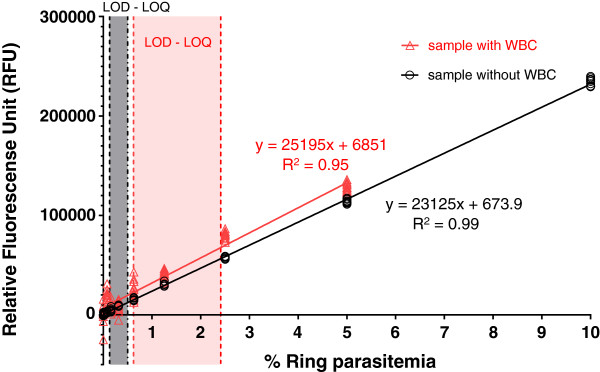
**SYBR Green I fluorescence linearity in *Plasmodium falciparum *ring stages of 0-10% parasitaemia.** Data points show average RFUs of 12 replicate wells, derived from one experiment using the *P. falciparum* K1 clone, plotted on the Y axis against % parasitaemia on the X axis. Red and black symbols, lines, and letters represent samples spiked with 8,500 WBC/μL and samples without WBCs, respectively. Regression line and R^2^ are shown. Dashed lines indicate LOD and LOQ range parasitaemia values obtained from up to three experiments.

The linearity of SYBR Green fluorescence in *P*. *falciparum* reference strain samples was compared with and without the addition of WBCs (Figure 1). For samples without WBCs, a range of MSF assay LOD and LOQ values of 0.13-0.15% and 0.43-0.50% parasitaemia, respectively, was determined from the results of three independent experiments using either *P*. *falciparum* 3D7 or K1 reference clones. In contrast, the MSF assay appears to lose sensitivity in samples spiked with WBCs, as evidenced by higher LOD and LOQ values of 0.62/0.72% and 2.06/2.41% parasitaemia, respectively, from two independent experiments.

### HRP-2 and MSF assay comparisons

#### *In vitro* drug susceptibility testing

**Figure 2:**
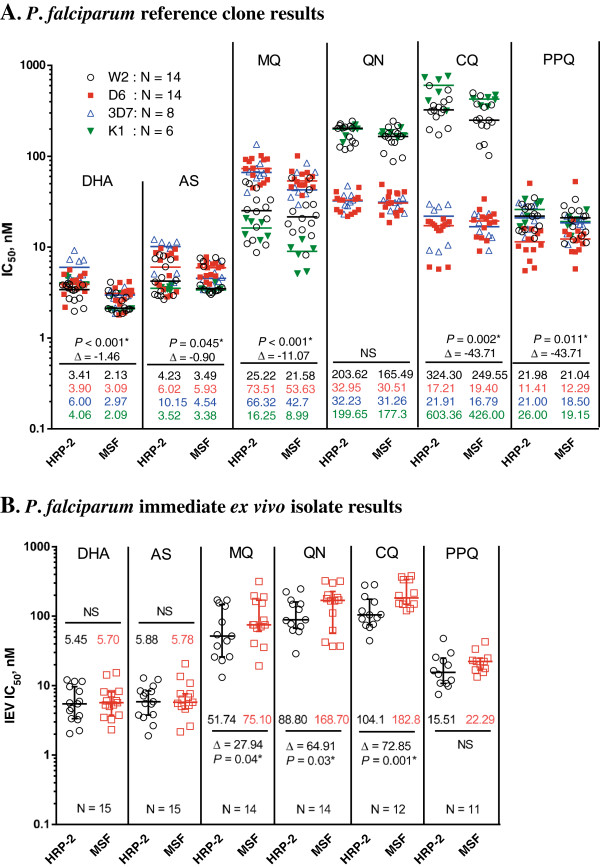
**Direct comparison of IC**_**50**_** values obtained using the HRP-2 and MSF assays. (A)** IC_50_s of reference laboratory clones (WBCs absent). Median IC_50_ values for each drug against W2, D6, 3D7 and K1 strains are indicated using different symbols/colours (n = number of IC_50_ values attained for each strain from 3–5 experiments). **(B)** IEV IC_50_s of field isolates (WBCs present). Of the total 41 IEV isolates evaluated in both the HRP-2 and MSF assays, IC_50_ values were obtained for both assays in 15 isolates. Median IC_50_ values for field isolates obtained by HRP-2 and MSF assays are shown as black circles and red squares, respectively. Significant *p*-values of Wilcoxon matched pair tests comparing IC_50_s from HRP-2 and MSF assays and their mean differences are designated with * symbol. NS signifies no statistical significance.

**Figure 3:**
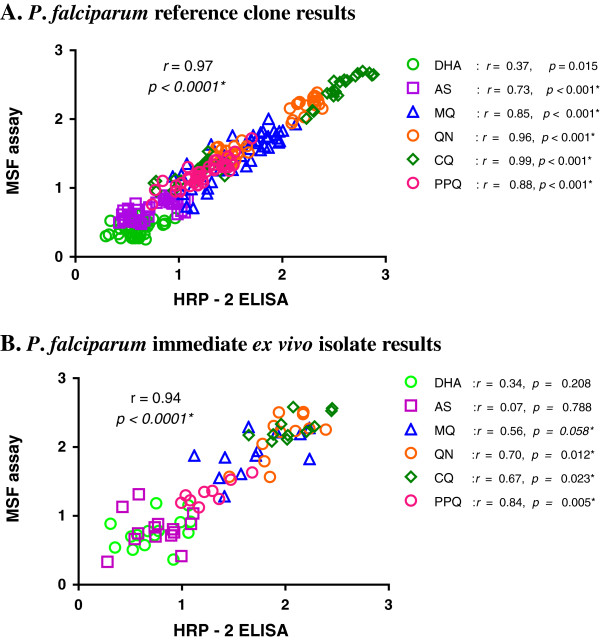
**Correlations between HRP-2 and MSF assay log [IC**_**50**_**], nM, results for reference strains and field isolates. (A)** IC_50_s of reference laboratory clones (WBCs absent). **(B)** IEV IC_50_s of 15 field isolates (WBCs present). Scatter plots are shown for the correlation of log [IC_50_s] of all drugs tested by HRP-2 and MSF assays. *P* value and *r* from Pearson correlation are shown for all drugs together and each drug tested individually. Significant correlations are designated with * symbol.

**Figure 4:**
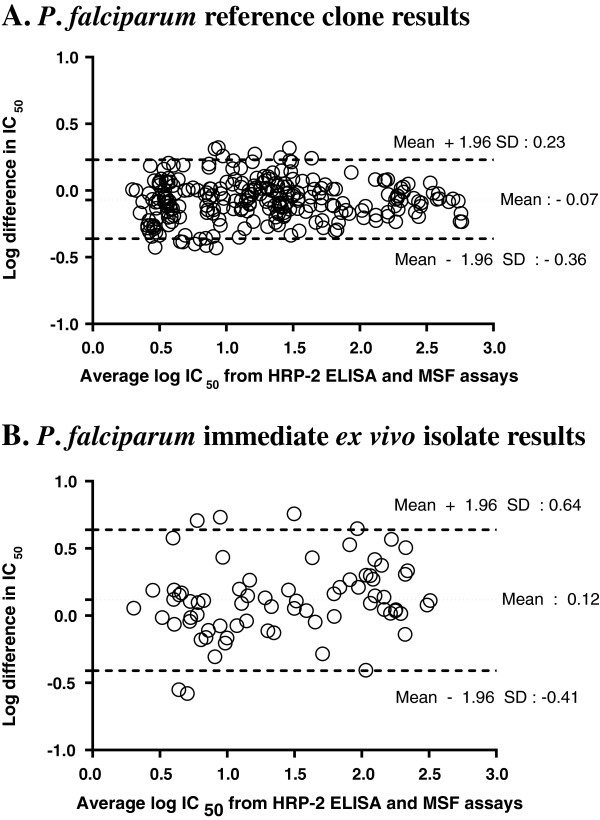
**Bland-Altman plots of the difference in log IC**_**50**_**s comparing HRP-2 and MSF assays. (A)** Bland-Altman analysis results for reference laboratory clones (WBCs absent). **(B)** Results for 15 IEV isolates (WBCs present) in which IC_50_ values were obtained for both assays.

The HRP-2 and MSF assays were evaluated simultaneously using a 0.5% baseline parasitaemia of *in vitro P*. *falciparum* reference strains with varying drug susceptibility profiles: W2 and K1 (susceptible to mefloquine, and resistant to chloroquine), D6 (susceptible to chloroquine, and resistant to mefloquine), and 3D7 (susceptible to chloroquine, but less susceptible to mefloquine than W2). From the IC_50_ profiles of all parasite laboratory strains (Figure 2A), both HRP-2 and MSF assays consistently differentiated sensitive from resistant strains against MQ, QN and CQ. Although overall IC_50_ values obtained from the HRP-2 ELISA were slightly higher than those from the MSF method (Figure 2A), significant correlations between both methods were found in all drugs tested (*r* = 0.73-0.99), except for DHA (Figure 3A). Analysis of pooled IC_50_s of all drugs tested revealed that both methods were significantly correlated (*r* = 0.97, *p < 0.0001*, Figure 3A). A Bland-Altman plot of IC_50_ values for all drugs is shown in Figure 4A, indicating a bias of −0.07 log units and limit of agreement between the two methods from −0.36 to 0.23 in log scale, further suggesting that results produced from both assays are comparable when testing *in vitro* reference strains.

#### Immediate *ex vivo* drug susceptibility testing

A total of 41 fresh *P*. *falciparum* isolates were analysed for IEV drug susceptibility using the HRP-2 and MSF assays in parallel. Success rate, defined as the proportion of samples in which it is possible to determine an IC_50_ value from a sigmoidal curve, appears to be dependent on baseline parasitaemia, as shown in Table 1. The HRP-2 ELISA demonstrated a high success rate (90%), whereas the MSF method was successful at determining IC_50_s in only 40% of samples. Of the total isolates tested, IC_50_ values were determined for 15 isolates using both assays. These 15 paired IC_50_ comparisons demonstrated consistently lower IC_50_s obtained from the HRP-2 ELISA than those from the MSF method for MQ, QN, and CQ (Figure 2B). Significant correlations between IC_50_s obtained from the two methods were found in all drugs tested, *r* = 0.56-0.84, except for DHA and AS, and good correlation was found for pooled IC_50_s against all drugs (*r* = 0.94, *P < 0.0001*, Figure 3B). A Bland-Altman plot of IC_50_s for all drugs tested is shown in Figure 4B, indicating a bias of 0.12 and limit of agreement between two methods from −0.41 to 0.64 in log scale, indicating tight correlation between the two methods when evaluating field isolates.

**Table 1:** **Success rates for HRP-2 and MSF assays in fresh *Plasmodium falciparum *isolates of varying parasitaemia**

**% parasitaemia**	**Success case (% success rate)**		**Total cases**
	**HRP-2 ELISA**	**MSF assay**	
<0.02%	1 (50%)	0 (0%)	2
0.02 - <0.2%	13 (87%)	0 (0%)	15
≥0.2%	23 (96%)	15 (63%)	24
Overall success	37 (90%)	15 (37%)	41

A total of 41 IEV *Plasmodium falciparum* isolates from Cambodia were evaluated in both the HRP-2 and MSF assays to determine % assay success rate.

#### Reliability of HRP-2 and MSF assays in samples with low parasitaemia

Two independent experiments were performed using W2 and D6 parasites to determine the lowest parasitaemia capable of producing sigmoidal concentration-response curves to determine IC_50_ values using the HRP-2 and MSF assays. Samples with a parasitaemia of 0.005, 0.01, 0.05, 0.2, and 0.5% were tested for *in vitro* drug susceptibility. For HRP-2 ELISA, a valid IC_50_ value was determined with minimal baseline parasitaemia as low as 0.005% or 0.05%, depending on the rate of parasite growth during 72 hours’ incubation. In contrast, the MSF assay requires an approximately one order of magnitude higher baseline parasitaemia range of 0.01-0.2%, relative to direct comparison using HRP-2 ELISA in the same samples, to produce IC_50_ values.

Similarly, when evaluating IEV isolates, the MSF overall success rate (37%) was lower than for HRP-2 ELISA (90%). The MSF assay required ≥0.2% baseline parasitaemia for success, whereas the HRP-2 assay can produced results from isolates with parasitaemia <0.2% (Table 1).

#### Effect of % parasitaemia on IC_50_ values

**Figure 5:**
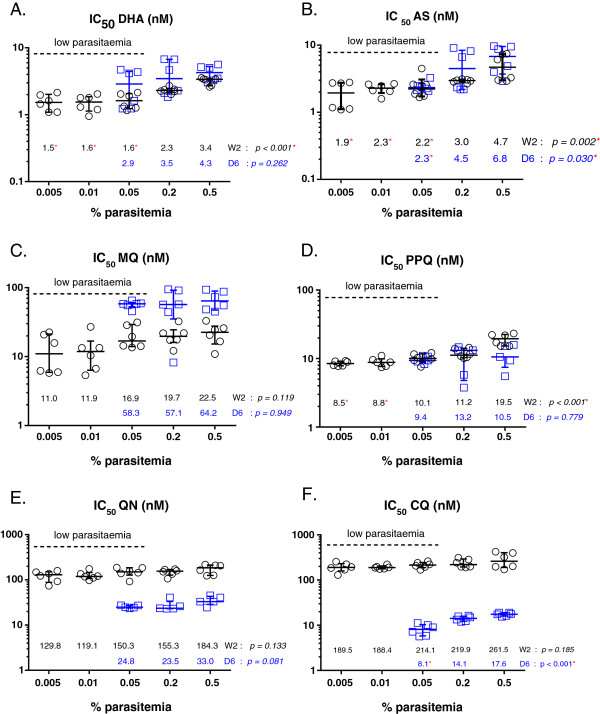
**Effect of baseline parasitaemia on IC**_**50**_** values for drugs tested by HRP-2 ELISA. (A)** DHA, **(B)** AS, **(C)** MQ, **(D)** PPQ, **(E)** QN, and **(F)** CQ. Results presented are IC_50_ values for drugs against W2 and D6 strains obtained from 2 independent experiments. The Kruskal–Wallis test was utilized to compare IC_50_ values when testing a range of parasitaemias from 0.005 to 0.5% for W2 parasites and from 0.05 to 0.5% for D6 parasites. Red stars indicate significant *p*-values and reduced IC_50_ values compared to those attained from a 0.5% parasitaemia baseline (*p* < 0.05 by Dunn’s Multiple comparison). Black circles and blue squares indicate IC_50_ values against W2 and D6, respectively. The dashed line in each graph indicates low baseline parasitaemia (<0.2%).

The effect of low baseline parasitaemia applied to the HRP-2 assay was evaluated because lower parasitaemia samples could produce an ‘inoculum effect’, a potential source for drug sensitivity test result variability resulting in lower IC_50_ values produced with lower baseline parasite density [24]. Low baseline parasitaemia for *P*. *falciparum* W2 and D6 reference strains of 0.005, 0.01, 0.05, 0.2, and 0.5% were tested in the HRP-2 assay in 2 independent experiments. This range of parasitaemia was chosen because it is representative of parasite densities encountered in Cambodian clinical isolates [9]. IC_50_ values were attained for all parasite densities tested for W2, whereas a parasitaemia <0.05% did not yield IC_50_ results for D6 (Figure 5). The results suggest that for all drugs tested an inoculum effect was observed to a certain extent, yielding higher IC_50_ values with increasing parasitaemia. For instance, an inoculum effect was observed as statistically significant lower IC_50_ values for DHA and AS when evaluating samples with a low starting parasitaemia (<0.2%) of *P*. *falciparum* W2 and D6 lines compared to higher parasitaemia samples (Figure 5A, 5B). For PPQ, significantly lower IC_50_ values were attained for a W2 baseline parasitaemia <0.05% (Figure 5D). For CQ, an inoculum effect appears more obvious in the CQ-resistant W2 line relative to the CQ-sensitive D6 strain (Figure 5F). Likewise, a more noticeable inoculum effect is noted for MQ in the MQ-resistant D6 strain than in the MQ-susceptible W2 clone (Figure 5C).

## Discussion

This is the first study to report the direct comparison of two field applicable methods, the HRP-2 and MSF assays, for evaluating drug resistance trends in immediate *ex vivo* isolates from Southeast Asia. Although several studies demonstrate successful use of the MSF assay for evaluating *P*. *falciparum* isolates, all of these investigations used relatively high parasitaemia samples with mostly ≥0.2% parasitaemia from Africa [6,17,25]. However, results reported here using 41 fresh *P*. *falciparum* field isolates from Cambodia suggest that the HRP-2 ELISA offers superior sensitivity, compared to the MSF assay, in lower parasitaemia samples (<0.2%) typical of malaria infections endemic to Southeast Asia. For instance, nearly half (49%) of clinical isolates collected from prior drug resistance surveillance work in Cambodia in 2009–2012 were <0.2% parasitaemia (AFRIMS data, n = 209 isolates). In contrast, only 22% of field isolates collected from Kenya in 2008–2009 were <0.2% parasitaemia (data from Kenya Medical Research Institute-Walter Reed Project, Kisumu, n = 292 isolates). This discrepancy in the success of the two methods in clinical isolates appears to result from reduced sensitivity of the MSF assay in lower parasitaemia samples containing WBCs (Figure 1).

Good correlation of IC_50_ values for MQ, QN, CQ, and PPQ was observed when comparing HRP-2 and MSF assay results, but it is unclear why poor correlation between methods was determined for the artemisinins (Figure 3). One possibility relates to assay reliability linked to mode and timing of drug action, since consistency of results for anti-malarial drug susceptibility assays depends on these drug-dependent variables [26]. Differences in methods for measuring parasite growth could possibly result in varying IC_50_ results. For instance, SYBR Green I fluorescence measures DNA replication and thus is less likely to produce results biased on parasite growth stage [27], whereas the HRP-2 protein is relatively stable and is actively secreted during parasite maturation stages [28]. However, lack of a true artemisinin-resistant reference strain hinders a meaningful comparison of methods. Unlike artemisinins, *P*. *falciparum* reference clones and field isolates are available that are resistant or sensitive to MQ, CQ and QN, allowing for robust comparisons of drug susceptibility assays [21]. In the absence of a standard artemisinin-resistant strain, further evaluation with more field isolates is needed to understand the sensitivities of the HRP-2 and MSF methods for differentiating artemisinin susceptibility from resistance. However, it is highly likely that *in vitro* assays will not detect resistance to fast-acting drugs, such as the artemisinins, and delayed parasite clearance time and rate may continue to be the gold standard means for detecting artemisinin resistance [29,30].

Besides considering mechanism of drug action, the effect of baseline parasitaemia is another variable to consider when interpreting drug sensitivity assay results from clinical isolates. In the HRP-2 assay, a significant reduction in DHA and AS IC_50_ values was observed when testing samples with a low starting parasitaemia (<0.2%) of *P*. *falciparum* W2 and D6 lines relative to higher parasite densities (Figure 5). This finding has been noted previously in a study reporting higher artemisinin IC_50_ values, and thus an overestimation of artemisinin resistance, associated with an inoculum effect in Gambian field isolates [24]. Interestingly, for CQ, an inoculum effect was only noted in the CQ-sensitive D6 line, whereas baseline parasitaemia did not appear to alter HRP-2 assay results in the CQ-resistant W2 strain (Figure 5). A difference in the chloroquine cellular accumulation ratio between chloroquine-resistant and chloroquine-sensitive strains could explain these findings. In particular, kinetic studies suggest a faster rate of chloroquine accumulation in chloroquine-sensitive parasites and greater extent of drug being depleted from the cellular growth medium [31], which could result in greater concentrations of drug (i.e., higher IC_50_ values) required to kill chloroquine-sensitive strains with increases in inoculum size [32,33]. Overall, these results suggest that lower baseline parasitaemias reduce HRP-2 IC_50_ values when evaluating highly susceptible, rather than drug-resistant, parasites.

Thus, it is possible that IC_50_ values could be lower for the artemisinins against susceptible clinical isolates of lower parasitaemias (<0.2%). Indeed, in comparing HRP-2 assay IC_50_ results attained with parasitaemia <0.2% *versus* ≥0.2% in a total of 350 IEV isolates from Cambodia collected in 2009–2012, significantly higher DHA and AS IC_50_ values were observed in samples with ≥0.2% parasitaemia (median IC_50_ of 8.2 for DHA and 6.5 for AS) compared to those attained from isolates with <0.2% parasitaemia (median IC_50_ of 6.2 for DHA and 4.1 for AS). However, interpretation of these results is complicated since isolates represent a mix of parasite populations, with potentially varying degrees of artemisinin susceptibility. To avoid potential confounding results, consistency in assay baseline parasitaemia was maintained to appropriately evaluate drug resistance surveillance results in field isolates [9].

The main conclusion of the present study is that HRP-2 ELISA is a more effective field-ready method, compared to the MSF assay, for analysing fresh clinical isolates of relatively low parasitaemia. However, as reported in this investigation and in previous reports [8,34], WBC depletion to decrease non-specific SYBR Green fluorescence I is expected to improve MSF assay sensitivity. Moreover, elimination of haemoglobin that also interferes with fluorescence readings was found to increase signal and assay sensitivity for low parasitaemia samples [34]. However, such additional sample processing steps to improve MSF assay performance in low parasitaemia isolates may ultimately be time consuming and labour intensive to justify practical use in the field. Since conducting this study, a modified version of the MSF assay [35] was developed involving a higher concentration of SYBR Green I dye and other modifications that could improve performance of the assay in field samples. Future efforts will investigate the effect of improving MSF assay performance with leucocyte depleted clinical isolates from Cambodia.

## Conclusions

In the context of clinical samples not processed for WBC depletion, HRP-2 ELISA is superior to the MSF assay at evaluating fresh *P*. *falciparum* isolates with low parasitaemia (<0.2%) typical of malaria endemic to Southeast Asia. Follow-on efforts are aimed at determining if performance of the MSF assay can be improved in fresh clinical isolates of low parasitaemia by removing WBCs to reduce non-specific SYBR Green I fluorescence. Ultimately, field deployable malaria drug sensitivity assays must be convenient, requiring minimal sample preparation and relatively simple methods, and produce reliable results suitable for monitoring drug resistance specific to the malaria-endemic region of interest.

### Endnote

The opinions and assertions contained herein are the private views of the authors and are not to be construed as official or as reflecting the views of the US Department of the Army. All human use research received the required ethical approvals from the appropriate authorities.

## Competing interests

The authors declare that they have no competing interests.

## Authors’ contributions

SC designed the study, conducted experiments, analysed and interpreted data and wrote the manuscript. SDT participated in study design, results interpretation, manuscript preparation, project conception and management. KY, SS and PS conducted experiments, collected and analysed data. WR and JDJ contributed to results interpretation and manuscript preparation. DSW and DLS contributed to project conception and management. CL participated in design of the study, conducted experiments, collected data and contributed to project conception and management. CAL participated in design of the study, interpreted data and wrote the manuscript. All authors read and approved the final manuscript.
